# Low-dose ^18^F-FDG TOF-PET/MR for accurate quantification of brown adipose tissue in healthy volunteers

**DOI:** 10.1186/s13550-020-0592-8

**Published:** 2020-01-23

**Authors:** Edwin E. G. W. ter Voert, Hanna Svirydenka, Julian Müller, Anton S. Becker, Miroslav Balaz, Vissarion Efthymiou, Claudia Irene Maushart, Gani Gashi, Christian Wolfrum, Matthias J. Betz, Irene A. Burger

**Affiliations:** 1 Department of Nuclear Medicine, University Hospital Zürich, Rämistrasse 100, 8091 Zürich, Switzerland; 2 University of Zurich, Rämistrasse 71, 8006 Zürich, Switzerland; 3 Department of Nuclear Medicine, Medical University Innsbruck, Anichstrasse 35, 6020 Innsbruck, Austria; 4 Department of Health Sciences and Technology, Institute of Food, Nutrition and Health, ETH Zürich, Schorenstrasse 16, 8603 Schwerzenbach, Switzerland; 5 Institute of Diagnostic and Interventional Radiology, University Hospital Zürich, Rämistrasse 100, 8091 Zürich, Switzerland; 6 Department of Endocrinology, Diabetes and Metabolism, University Hospital Basel, Petersgraben 4, 4031 Basel, Switzerland; 7 University of Basel, Petersplatz 1, 4001 Basel, Switzerland; 8 Department of Nuclear Medicine, Kantonsspital Baden, Im Ergel 1, 5404 Baden, Switzerland

**Keywords:** Brown adipose tissue, ^18^F-FDG, PET/MR, Dose optimization, Supraclavicular region, Clinical

## Abstract

**Background:**

Positron emission tomography (PET) is increasingly applied for in vivo brown adipose tissue (BAT) research in healthy volunteers. To limit the radiation exposure, the injected ^18^F-FDG tracer dose should be as low as possible. With simultaneous PET/MR imaging, the radiation exposure due to computed tomography (CT) can be avoided, but more importantly, the PET acquisition time can often be increased to match the more extensive magnetic resonance (MR) imaging protocol. The potential gain in detected coincidence counts, due to the longer acquisition time, can then be applied to decrease the injected tracer dose. The aim of this study was to investigate the minimal ^18^F-FDG dose for a 10-min time-of-flight (TOF) PET/MR acquisition that would still allow accurate quantification of supraclavicular BAT volume and activity.

**Methods:**

Twenty datasets from 13 volunteers were retrospectively included from a prospective clinical study. PET emission datasets were modified to simulate step-wise reductions of the original 75 MBq injected dose. The resulting PET images were visually and quantitatively assessed and compared to a 4-min reference scan. For the visual assessment, the image quality and artifacts were scored using a 5-point and a 3-point Likert scale. For the quantitative analysis, image noise and artifacts, BAT metabolic activity, BAT metabolic volume (BMV), and total BAT glycolysis (TBG) were investigated.

**Results:**

The visual assessment showed still good image quality for the 35%, 30%, and 25% activity reconstructions with no artifacts. Quantitatively, the background noise was similar to the reference for the 35% and 30% activity reconstructions and the artifacts started to increase significantly in the 25% and lower activity reconstructions. There was no significant difference in supraclavicular BAT metabolic activity, BMV, and TBG between the reference and the 35% to 20% activity reconstructions.

**Conclusions:**

This study indicates that when the PET acquisition time is matched to the 10-min MRI protocol, the injected ^18^F-FDG tracer dose can be reduced to approximately 19 MBq (25%) while maintaining image quality and accurate supraclavicular BAT quantification. This could decrease the effective dose from 1.4 mSv to 0.36 mSv.

## Background

Obesity and associated metabolic disorders are a fast-growing global health problem, having a significant impact on healthcare costs, and more importantly also on the quality of life [1]. Intensive research over the past years has changed the traditional view of adipose tissue from being an inert, lipid-storing depot to a highly dynamic, endocrine organ. The growing interest for a better understanding of brown adipose tissue (BAT) was triggered by the discovery of active BAT on ^18^F-fluoro-2-deoxy-D-glucose (^18^F-FDG) positron emission tomography (PET) scans in adult oncology patients [2].

Brown adipocytes contain, in contrast to white adipocytes, a very high amount of mitochondria, have multilocular lipid droplets, and contribute to thermogenesis by directly dissipating chemical energy as heat [3]. This process is facilitated by a specific channel (uncoupling protein 1, UCP1) located on the mitochondrial membrane, which can uncouple the respiratory chain from the regeneration of adenosine triphosphate (ATP) [4].

The high glucose consumption of activated BAT leads to a large number of studies, using ^18^F-FDG PET as a surrogate marker for its metabolic activity [5–7]. Most of these studies, looking into the prevalence and factors that correlated with high BAT activity, were done on retrospective data and showed that active BAT was more commonly seen on ^18^F-FDG PET scans in winter than in summer, in women than in man, and in normal weight vs obese patients [5, 8–12].

An increasing number of prospective trials, investigating the activity of BAT with ^18^F-FDG PET, were published in the last few years [13–15], however without any accepted standardization of the protocols [16]. Therefore, a suggestion on how to image and quantify BAT activity was published in 2016: the Brown Adipose Reporting Criteria in Imaging Studies (BARCIST 1.0) [17]. Additionally, to detailed recommendations regarding selection of volunteers, BAT activation (cooling or pharmaceutical), image acquisition, reconstruction, and quantification, the authors also noted that the injected dose of ^18^F-FDG should be as low as possible, for statistically valid imaging, with consideration for total dosage in repeat studies [17]. However, decreasing the injected ^18^F-FDG dose could lead to decreased PET image quality, more noise, and quantification errors.

One of the key elements for good PET image quality and reliable quantification is detecting sufficient coincidence events. Lowering the injected tracer dose means fewer positron-emitting radionuclides and thus less detected 511 KeV photon pairs. This count loss can to some extent be compensated by longer PET acquisition times. Longer scan time, however, increases discomfort and the risk of bulk motion. It also decreases efficiency as fewer scans can be performed in a day. Hence, an optimum has to be determined between injected tracer dose and PET acquisition time.

One of the advantages of a simultaneous PET/magnetic resonance (MR) scanner is that PET and MR acquisitions can be acquired at the same time. This means that the PET acquisition time can often be increased to match the more extensive magnetic resonance (MR) imaging protocol. The potential gain in detected coincidence counts, due to the longer PET acquisition time, can then be applied to decrease the injected tracer dose.

With MR several biomarkers for BAT, research can be investigated. A recent publication by Karampinos et al. gives a nice overview of several techniques and applications [18]. Common MR sequences often include, next to an 18-s acquisition time MR-AC sequence for PET attenuation, T1- and T2-weighted sequences (approximately 3 min and 30 s acquisition time, respectively) for anatomical reference. In-phase, out-of-phase, water-only, and fat-only image datasets are already generated by the MR-AC sequence, but sometimes more accurate versions are needed. The iterative decomposition with echo-asymmetry and least squares estimation (IDEAL)-IQ sequence (GE Healthcare, Waukesha, WI, USA), a three-dimensional gradient multi-echo sequence, for example, takes differences in T2* into account and generates six image datasets: in-phase, out-of-phase, water-only, fat-only, proton-density fat fraction (PDFF), and R2*(= 1/T2*) (approximately 3 min acquisition time) [19, 20]. Other biomarkers that can be investigated are diffusion and perfusion with, e.g., diffusion-weighted MR imaging (DWI), intravoxel incoherent motion (IVIM) imaging, dynamic contrast-enhanced MRI (DCE-MRI), arterial spin labeling (ASL), or blood-oxygen-level-dependent (BOLD) imaging [18]. Metabolic activities can be investigated, next to PET, with, e.g., ^1^H/^31^P/^13^C MR spectroscopy, although these can be more time-consuming [18].

As the commonly performed MR protocols (like T1-, T2-, and T2*-weighted; PDFF; and, e.g., DWI) require approximately 10 min, we defined this as the optimal PET scan duration for PET/MR. With simultaneous PET/MR imaging, the radiation exposure due to computed tomography (CT), as applied in hybrid PET/CT scanners, can of course also be avoided.

It was therefore the aim of our study to minimize the radiation exposure for healthy volunteers by finding the minimal ^18^F-FDG dose for a 10-min time-of-flight (TOF) PET/MR acquisition that would still allow accurate quantification of supraclavicular BAT volume and activity.

## Methods

### Participants and datasets

In this retrospective study, a total of 20 ^18^F-FDG TOF PET/MR datasets from 13 healthy male Caucasian volunteers (median age, 23 years; range, 19–28 years; median body mass index, 22.9 kg/m^2^; range, 18.6–25.4 kg/m^2^) were obtained from a prospective clinical study [21]. In that study, each subject received two PET/MR scans, separated by 14 days. However, only 20 datasets were available for retrospective reconstructions. In short, 4 h before the PET/MR scan, all participants received 200 mg mirabegron per os, a selective β_3_-adrenoreceptor agonist, to stimulate BAT activity. Two and a half hours before the scan, the volunteers were exposed to a standardized mild cooling protocol by applying two cooling sleeves around the subjects’ abdomen and chest, connected to a medical cooling device (Hilotherm Clinic®, Hilotherm GmbH, Germany). Next, the volunteers were scanned using a simultaneous TOF PET/MR system (SIGNA PET/MR, GE Healthcare, Waukesha, WI, USA), having a PET axial field-of-view (FoV) of 25 cm and a TOF timing resolution of 400 ps [22, 23]. After performing a partial body MR localizer scan, a 30-min dynamic three-dimensional (3D) PET acquisition of the neck area (one bed station) was started and 75 MBq of ^18^F-FDG was injected over an intravenous line. The dynamic PET scan was followed by a static 3D PET emission partial body scan. It consisted of three bed stations, each with a duration of 4 min, covering an area from the head to the upper abdomen. During PET scanning, a default 3D dual-echo, spoiled gradient recalled echo MR sequence was performed for PET attenuation correction (MR-AC). Fat and water images were automatically reconstructed from the obtained in-phase and out-of-phase images. The PET attenuation correction algorithm uses an atlas for the head region and a continuous fat-water-based attenuation correction method for the other body parts [24, 25]. Besides the MR-AC sequence, T_1_-, T_2_-, and T_2_*-weighted MR imaging was performed as well as diffusion weighted MR imaging (DWI).

### PET dose reductions

In this study only, the last 10 min of the 30-min PET frames were used for the simulated dose reductions. The minimal dose was expected to be below 40% of the original dose, as the 10-min scan is 2.5 times longer than the standard 4-min static scans used in this and other studies [21, 26]. Therefore, every 10-min dataset was reconstructed using seven dose reduction steps, having approximately 35%, 30%, 25%, 20%, 15%, 10%, and 5% of the original counts.

To do so, first, an artificial trigger signal was inserted every second in the PET list-mode datasets using an in-house developed Matlab script (MATLAB R2018a, MathWorks, Natick, MA, USA). Next, the scanner’s gating options were enabled which allowed us to select only those counts for PET reconstruction that were detected during the above-indicated percentage of time between each trigger signal. This way, the counts are reduced on a second by second basis, thereby simulating a corresponding lower injected dose. It also means that normal effects like decay, biodistribution, and possibly volunteer motion remain included. This unlisting process was repeated seven times for each PET list-mode dataset to generate the seven TOF PET sinograms with approximately the previously indicated counts, simulating a lower activity due to a reduced injected dose.

### PET reference datasets

The 4-min reference datasets applied in this research were created using the same 30-min PET frame datasets that were used for the 10-min reduced activity datasets. During PET reconstruction, the start time was set to 20 min to match the start time of the reduced activity datasets, and the duration was set to 4 min. This means that the “uptake” times of the reference and reduced activity reconstructions are the same.

### PET reconstructions

PET reconstructions were performed using the scanner’s three-dimensional ordered subset expectation maximization (3D-OSEM) based reconstruction algorithm for TOF PET data (VUE Point FX, GE Healthcare, Waukesha, WI, USA). PET reconstructions included all standard corrections like decay, scatter, random, dead time, attenuation, normalization, and the detector response. The number of subsets was 28 and the number of iterations was 2, the reconstruction diameter was 60 cm, and the image grid was 256 × 256 with 2.34 × 2.34 × 2.78 mm^3^ voxels. All OSEM reconstructions were post-filtered in image space using an in-plane Gaussian convolution kernel with a full-width-at-half-maximum of 5.0 mm, followed by a standard axial filter with a three-slice kernel using relative weights of 1:4:1.

### Image analysis

All images were analyzed on a dedicated workstation (Advantage Workstation 4.6, GE Healthcare), which allowed images to be viewed side-by-side as well as in fused mode. PET images were visually examined and scored in consensus by an experienced nuclear medicine physician and a dual trained experienced nuclear medicine physician and radiologist. The image quality was scored using a 5-point Likert scale (1, non-diagnostic image quality to 5, excellent image quality) and image artifacts were scored using a 3-point scale (0, no artifacts; 1, insignificant artifacts; 2, significant artifacts).

For the quantitative analysis, the standardized uptake values (SUL) were normalized using the lean body mass (LBM) [17, 27]. Cubic 16.6cm^3^ volumes of interest (VOIs) were drawn bilateral in a homogeneous part of the infraspinatus muscle and propagated to all other reconstructions with different activities of the same scan (Additional file 1: Figure S1). Maximum SUL (SUL_max_), mean SUL (SUL_mean_), and the standard deviation (SUL_std_) were obtained from each VOI. To assess the image background noise, the coefficient of variation (SUL_cov_ *=* SUL_std_/SUL_mean_) was calculated.

VOIs were also drawn around artifacts using the workstation autocontour tool. Only artifacts having a minimal SUL of 2.0 g/ml on the 5% activity reconstructions were included as these are expected to affect the reporting of BAT. The 5% activity reconstructions were chosen as those have the highest noise.

The effect of dose reduction on BAT metabolic activity, BAT metabolic volume (BMV), and total BAT glycolysis (TBG; counterpart to the normally used PET-metric “total lesion glycolysis TLG”) was also investigated [8]. For this, VOIs were drawn bilateral around supraclavicular BAT and SUL_max_ and SUL_mean_ were obtained. BMV was defined as the sum of all voxel volumes within the suspected BAT region where SUL > 1.2 g/ml. The TBG was defined as the product of the BMV and its corresponding SUL_mea_ n[17].

Percentage difference (%diff) parameters (*X*) were calculated using the 4-min datasets as reference: %diff = (*X*_*y*%_ − *X*_ref%_)/*X*_ref%_ × 100, where *y* is any of the reconstructions in the range 5–35%.

### Statistical analysis

Statistical analysis was performed using Prism 7 (GraphPad Software Inc., San Diego, California, USA). Differences in the visual and quantitative parameters were assessed using the Friedman test followed by Dunn’s test. Results were considered statistically significant when *p* < 0.05.

## Results

**Fig. 1:**
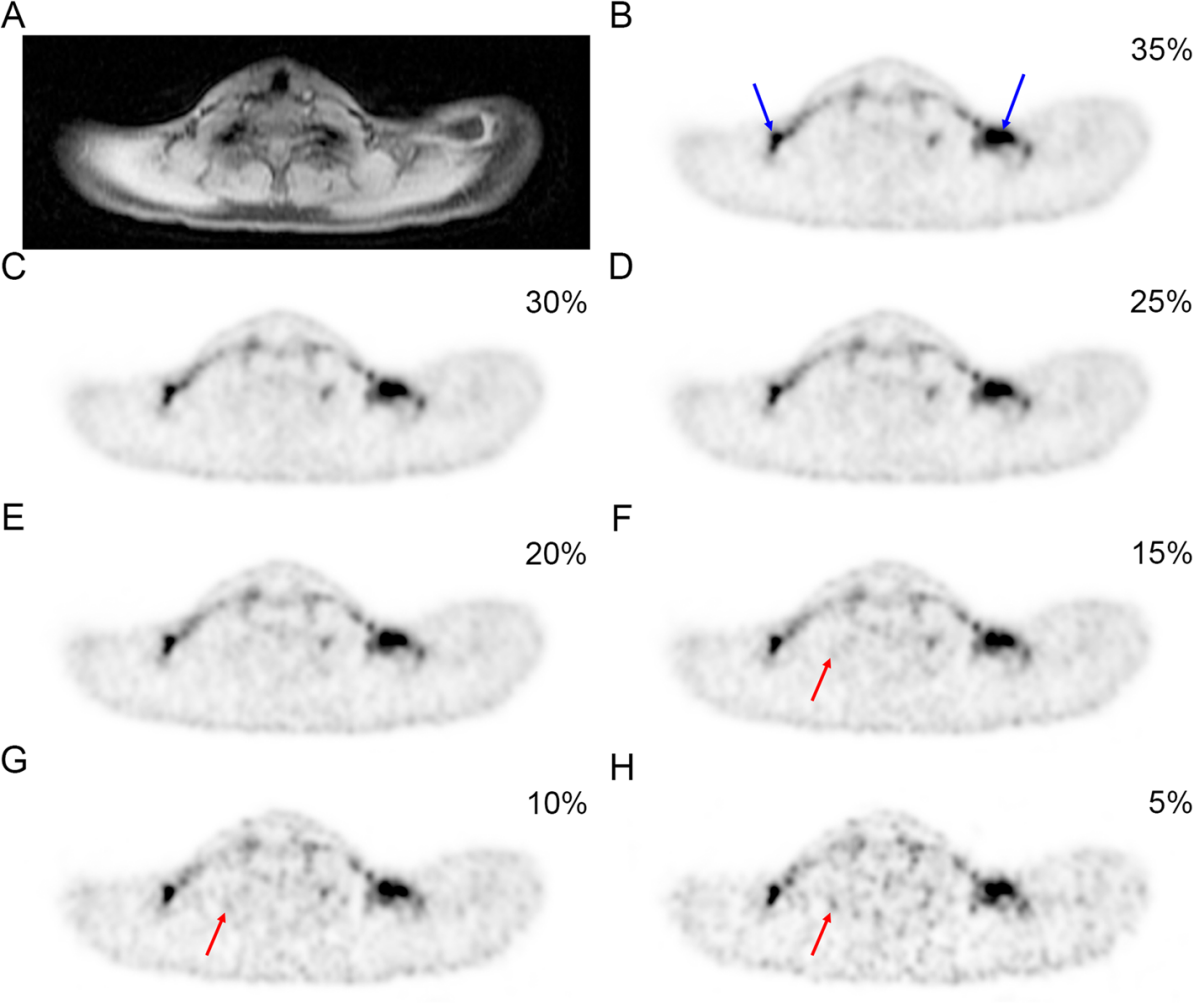
Representative dataset showing the supraclavicular BAT on MR and on PET reconstructed images with decreasing activity. **a** Axial MRI “water” image. **b**–**h** Axial TOF PET images reconstructed with 35%, 30%, 25%, 20%, 15%, 10%, and 5% of the original counts, thereby simulating corresponding lower doses. Blue arrows indicate supraclavicular BAT, and red arrows indicate increasing artifacts with decreasing activity

The 10-min PET acquisitions with 100% activity had a median “total counts” of 111 × 10^6^ (range 73–127 × 10^6^). The 10-min 35% and 5% activity reconstructions have therefore approximately 37 × 10^6^ and 5.3 × 10^6^ counts, respectively. Representative examples of 35% to 5% activity reconstructions showing supraclavicular BAT (blue arrows) are presented in Fig. 1. The axial images clearly show a decreasing image quality and an increasing amount of noise and artifacts (red arrows) with decreasing activity.

### Visual assessment

**Fig. 2:**
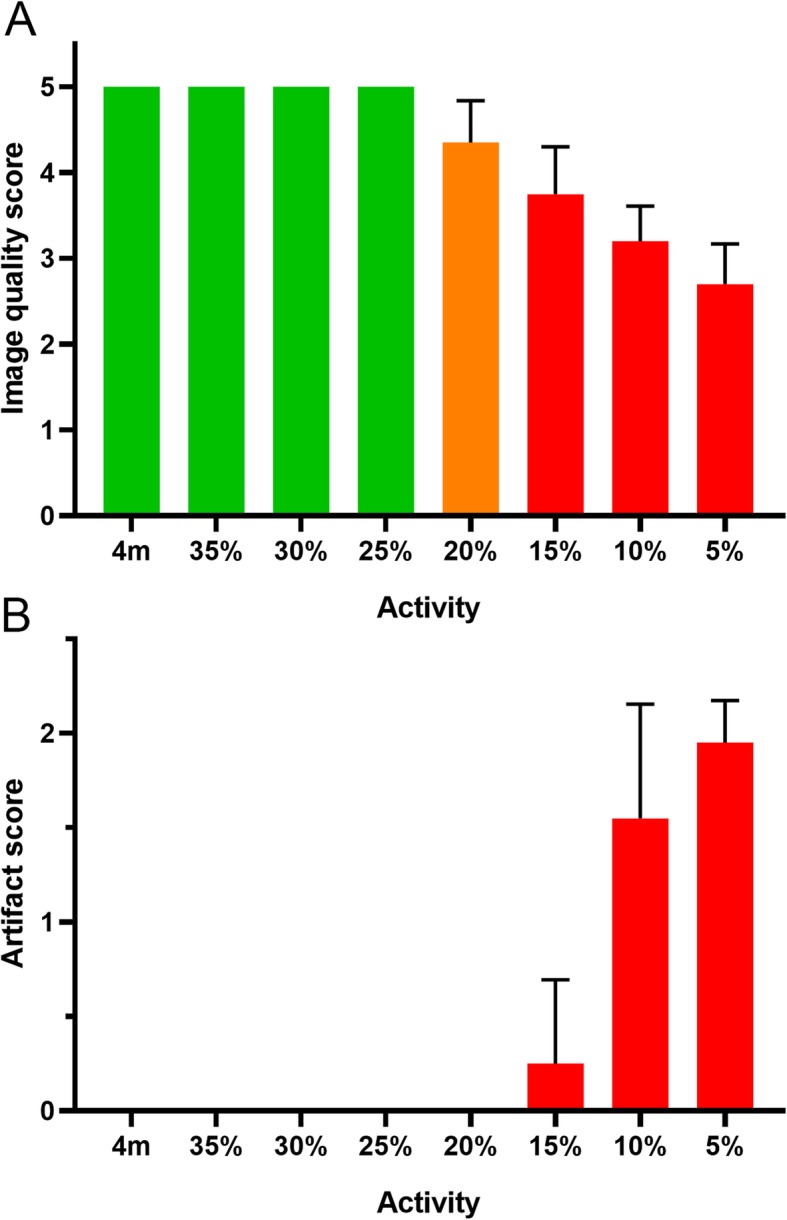
The image quality and artifact score for the reference and multiple simulated activities. The image quality score (**a**) shows that the 4-min reference reconstruction (4 m) and the reconstructions with 35%, 30%, and 25% of the original activity have a good image quality (green). The 20% reconstructions have a somewhat lower quality (orange). The artifact score (**b**) shows that reconstructions with 15%, 10%, and 5% activity have increasing artifacts (red). Results are reported as mean ± SD

The visual assessment showed that the reference and the 35%, 30%, and 25% activity reconstructions have good image quality (Fig. 2a) and no artifacts (Fig. 2b). Although the 20% activity reconstructions have a statistically significant reduction (*p* = 0.02) in image quality compared to the reference, the image quality was still acceptable with no artifacts. The 15%, 10%, and 5% activity reconstructions have a significantly lower (*p* < 0.01) image quality. Moreover, the 10% and 5% activity reconstructions also suffer from a significant increase (both *p* < 0.01) in artifacts and are therefore not recommended.

### Quantitative assessment for background and artifacts

**Fig. 3:**
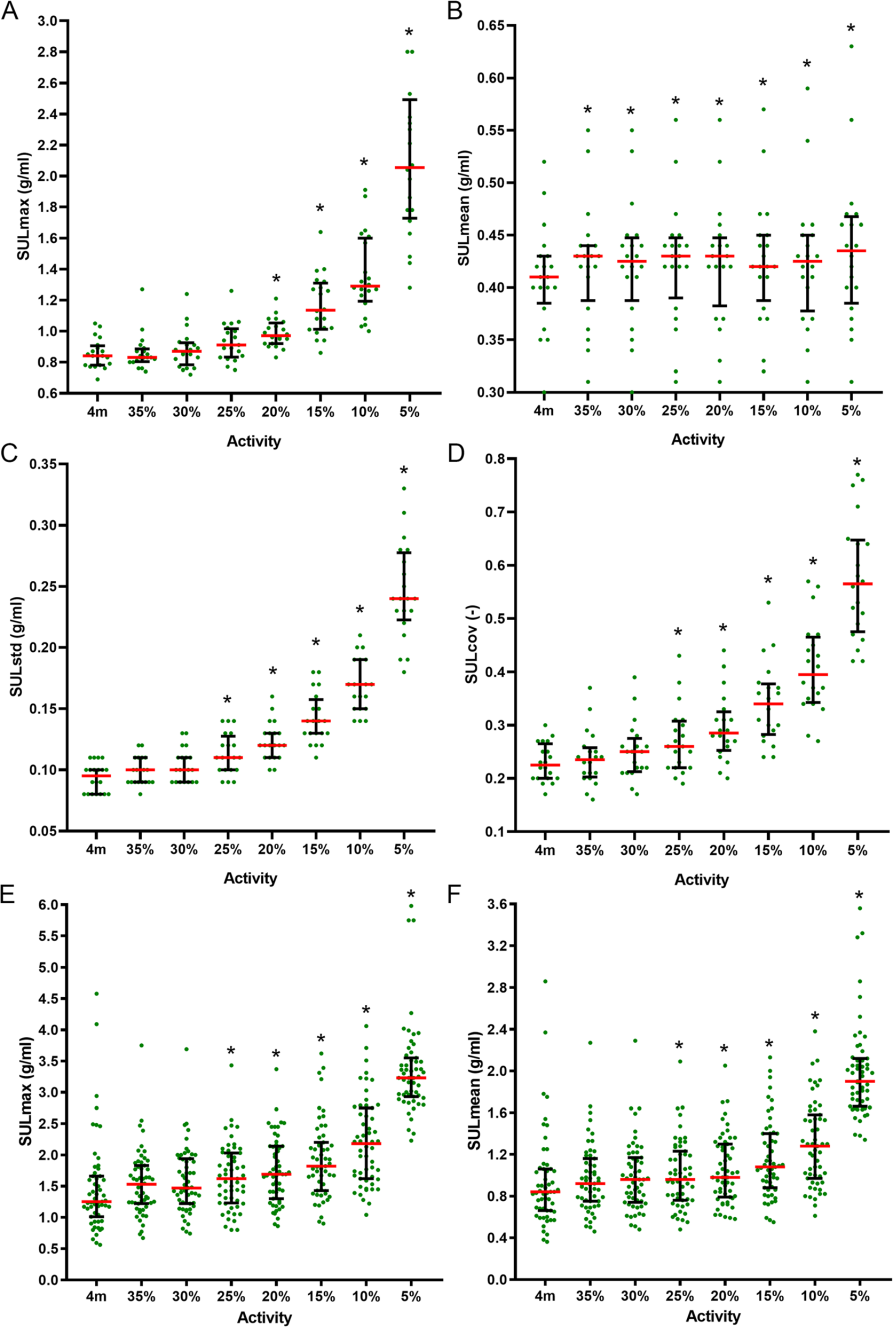
The measured background and artifact SUL values for the reference and multiple simulated activities. The SUL_max_, SUL_mean_, SUL_std_, and SUL_cov_ of the background VOIs are shown in **a**–**d**, respectively. The SUL_max_ and SUL_mean_ of the artifact VOIs are shown in **e** and **f**. The horizontal axis indicates the 4-min reference (4 m) and decreasing activity (*x*%) reconstructions. Results are reported as median ± interquartile range. An asterisk indicates a significant difference with the reference. For the 5% activity, 2 data points in the SUL_max_ graph (**a**) and 1 data point in the SUL_std_ graph (**c**) are outside the shown range

The SUL_max_, SUL_std_, and SUL_cov_, obtained from VOIs in the homogeneous part of the infraspinatus muscle, increase with decreasing activity (Fig. 3 and Additional file 1: Table S1). There was no significant difference in background SUL_max_ between the reference and the 35%, 30%, and 25% activity reconstructions. The 20% and lower activity reconstructions started to be significantly different from the reference (all *p* < 0.01). The background SUL_mean_ was significantly higher (*p* = 0.02 for 35% and 30%, *p* < 0.01 for 25–5%) in all reduced activity reconstructions, compared to the reference. There was no significant difference in background SUL_std_ between the reference and the 35% and 30% reconstructions. The 25% and lower reconstructions were all significantly different to the reference (all *p* < 0.01). The background noise (SUL_cov_) was previously defined as the ration between SUL_std_ and SUL_mean_. As the SUL_mean_ is approximately stable over the reduced activity range, the SUL_cov_ has similar results as the SUL_std_. This means that from a quantitative perspective, the noise started to increase at the 25% and lower activity reconstructions (all *p* < 0.01).

There was no significant difference in artifact SUL_max_ or SUL_mean_ between the reference and the 35% and 30% reconstructions (Additional file 1: Table S2). Artifact SUL_max_ or SUL_mean_ started to increase at the 25% and lower activity reconstructions (all *p* < 0.01)

### Quantitative assessment of supraclavicular BAT

**Fig. 4:**
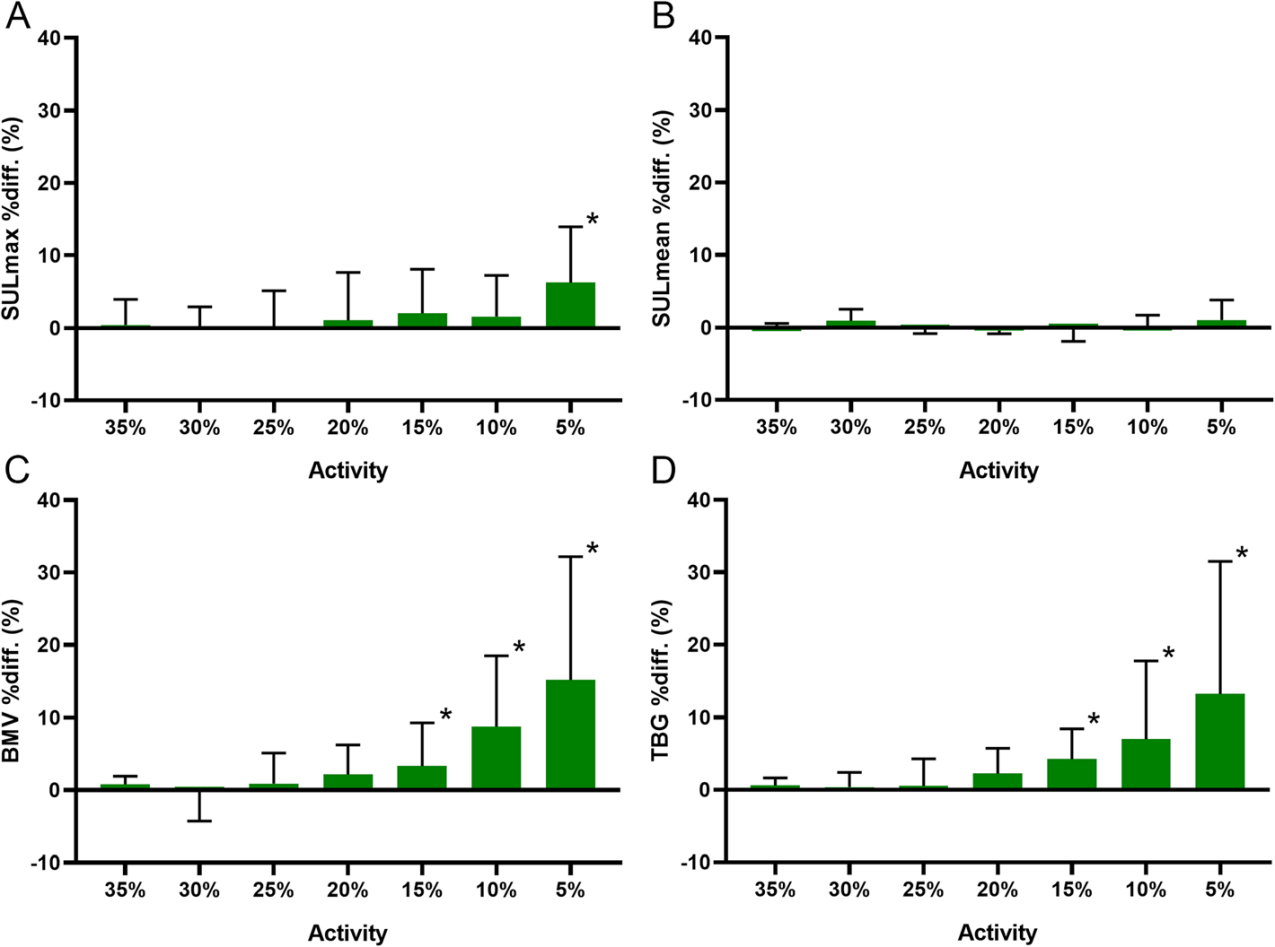
Supraclavicular BAT values for multiple simulated activities. The graphs show the SUL_max_ (**a**), SUL_mean_ (**b**), BAT metabolic volume (BMV) (**c**), and total BAT glycolysis (TBG) (**d**) percentage difference (%diff.) values in relation to the corresponding 4-min reference scan. Results are reported as median ± interquartile range. An asterisk indicates a significant difference with the reference

For supraclavicular BAT SUL_max_, only the 5% activity reconstructions have a significantly higher value compared to the reference (*p* < 0.01) (Fig. 4 and Additional file 1: Table S3). There was no significant difference in supraclavicular BAT SUL_mean_ between the reference and the reduced activity reconstructions. There is no significant difference in supraclavicular BAT BMV and TBG between the reference and the 35%, 30%, 25%, and 20% activity reconstructions. The 15%, 10%, and 5% activity reconstructions have increasing differences compared to the reference (*p* = 0.02, *p* < 0.01, *p* < 0.01, respectively, for both BMV and TBG).

Combining the information from the visual assessment, the quantitative assessment of the background noise and artifacts, and the quantitative assessment of supraclavicular BAT, we can see that the lowest possible dose would be 25% of the original dose. Although the artifacts and the background noise of the 25% activity reconstruction were significantly higher in the quantitative analysis, it was visually still evaluated as having good image quality.

## Discussion

Limiting the radiation exposure for volunteers in BAT studies using ^18^F-FDG PET is especially important when performing repeated scans and/or scanning of young individuals. Using a PET/MR instead of a PET/CT already lowers the radiation exposure due to the absence of exposure to X-rays. Another important factor for the radiation exposure is the injected ^18^F-FDG radiotracer dose. The recently published criteria (BARCIST 1.0) recognize this and recommend “using a dose as low as possible for statistically valid imaging, with consideration for total dosage in repeat studies” [17].

Overall, our study showed that with the 10-min PET frames, the injected dose can be reduced to 19 MBq (25%, approximately 28 × 10^6^ counts) compared to the already established dose of 75 MBq for 4 min scans, without a significant change in supraclavicular BAT metabolic volume and activity. Quantitatively, the background noise and artifacts increased, but these were not considered relevant in the visual assessment. An injected tracer dose of 19 MBq corresponds to an effective dose of only 0.36 mSv. As a comparison, commonly applied doses for BAT investigations in (healthy) adults range between 74 MBq and 444 MBq for PET/CT [12, 13, 15, 28] and PET/MR [29, 30].

With recent technical developments like the introduction of silicon photomultiplier (SiPM) based photodetectors in PET, the sensitivity and TOF resolution improved considerably. The benefits of these new systems often include improved image quality, higher resolution, more accurate quantifications, higher signal-to-noise ratios, and/or shorter scan times. These technical improvements can, on the other hand, also be used to reduce the injected radiotracer dose and thereby the radiation exposure for patients or healthy volunteers. Especially when scanning healthy, often young volunteers for research when there is no direct benefit for the participants, the injected dose should be kept as low as possible.

Recently published BAT studies already show that subjects are currently given a relatively low 75–80 MBq ^18^F-FDG dose for a PET scan resulting in an effective dose of approximately 1.4 mSv. When combined with a low-dose CT for attenuation correction and anatomical reference, this would result in a total effective dose of approximately 3.6 mSv [26]. However, with the current study, we indicate that significant reductions in radiation exposure are still possible while maintaining image quality when using PET/MR where the PET scan could efficiently be combined with multiparametric MRI.

It should be noted that the presented results depend on the detected number of coincidence counts. Therefore, higher doses are recommended for less sensitive PET scanners, shorter acquisition protocols, or, e.g., obese participants.

The current BARCIST guidelines recommend an uptake time of 60 min. This is based on previous experience with ^18^F-FDG in oncological imaging as the optimal uptake time for BAT research was not investigated before. For comparison reasons, it is best to use the same uptake time in all BAT research. In our research, we used a simulated uptake time of 20 min for both the reference and the reduced activity datasets.

A PET scanner detects prompt coincidences, which consist of true (what we want), scatter and random coincidences. At low activities, the detector response is approximately linear with increasing activity. At higher activities, detector saturation effects can start to paralyze the system and reduce performance such that the count rate is no longer linear with increasing activity. At low activities and in the normal clinical range, the true and scatter coincidences scale roughly linear with the activity (within limits), while the randoms scale more like a quadratic function (actual curves can be found in [31]). In a real low-dose PET scan, the amount of randoms is therefore likely lower than in our simulated low-dose PET. Hence, our simulated low-dose scans may somewhat underestimate the image quality.

A potential limitation of our study is that it included only male volunteers. Further studies should be performed to investigate the effect of dose reduction in female volunteers. To validate the results, future BAT studies could stepwise lower the injected dose.

## Conclusions

This study indicates that when the PET acquisition time is matched to a 10-min MRI protocol in simultaneous PET/MR, the injected ^18^F-FDG tracer dose can be reduced to approximately 19 MBq (25%) while maintaining image quality and accurate supraclavicular BAT quantification. This means for the participants, a decreased effective dose is from 1.4 mSv to 0.36 mSv.

## Supplementary information

**Additional file 1: Figure S1.** Examples of VOIs. (A) A coronal PET image showing two VOIs containing BAT, (B) a coronal fused PET/MR image showing the BAT, (C) an axial PET image showing two VOIs containing BAT, (D) an axial fused PET/MR image showing the BAT, (E) an axial fused PET/MR image showing VOIs for background measurements, (F) an axial 5% activity PET image showing a VOI containing an artifact, fused with Dixon based water (G) and fat (H) MR images. The colorbars indicate the SUL range (0-8 g/ml and 0-4 g/ml). **Table S1.** Background quantification. **Table S2.** Artifact quantification. **Table S3.** BAT quantification

## Data Availability

The datasets used and/or analyzed during the current study are available from the corresponding author on reasonable request. Sharing data could require approval of the cantonal ethics committee and written informed consent from each volunteer for retrospective use of their data.

## Abbreviations

%diff: Percentage difference
^18^F-FDG: Glucose analog tracer labeled with fluorine-18, (2-deoxy-2-(^18^F)fluoro-D-glucose)
3D: Three dimensional
AC: Attenuation correction
ASL: Arterial spin labeling
ATP: Adenosine triphosphate
BARCIST: Brown Adipose Reporting Criteria in Imaging Studies
BAT: Brown adipose tissue
BMV: BAT metabolic volume
BOLD: Blood-oxygen-level-dependent
CT: Computed tomography
DCE-MRI: Dynamic contrast-enhanced MRI
DWI: Diffusion weighted MR imaging
FoV: Field-of-view
IDEAL-IQ: Iterative decomposition with echo-asymmetry and least squares estimation
IVIM: Intravoxel incoherent motion
MR: Magnetic resonance
OSEM: Ordered subset expectation maximization
PCa: Prostate cancer
PDFF: Proton-density fat fraction
PET: Positron emission tomography
SiPM: Silicon photomultiplier
SUL: Standardized uptake value, normalized using the lean body mass
SULcov: Coefficient of variation of SUL
SULmax: Maximum of SUL
SULmean: Mean of SUL
SULstd: Standard deviation of SUL
SUV: Standardized uptake value
TBG: Total BAT glycolysis
TOF: Time-of-flight
UCP1: Uncoupling protein 1
VOI: Volume of interest
*X*: Parameter
