# Supplementary material for: Low-dose 18F-FDG TOF-PET/MR for accurate quantification of brown adipose tissue in healthy volunteers
Source: EJNMMI Res. 2020 Jan 23;10:5. doi: 10.1186/s13550-020-0592-8 (PMC6977803; doi:10.1186/s13550-020-0592-8)
Supplement: Supplementary file 1 — Additional file 1: Figure S1. Examples of VOIs. (A) A coronal PET image showing two VOIs containing BAT, (B) a coronal fused PET/MR image showing the BAT, (C) an axial PET image showing two VOIs containing BAT, (D) an axial fused PET/MR image showing the BAT, (E) an axial fused PET/MR image showing VOIs for background measurements, (F) an axial 5% activity PET image showing a VOI containing an artifact, fused with Dixon based water (G) and fat (H) MR images. The colorbars indicate the SUL range (0-8 g/ml and 0-4 g/ml). Table S1. Background quantification. Table S2. Artifact quantification. Table S3. BAT quantification [file 13550_2020_592_MOESM1_ESM.docx]

**Supplemental Information**


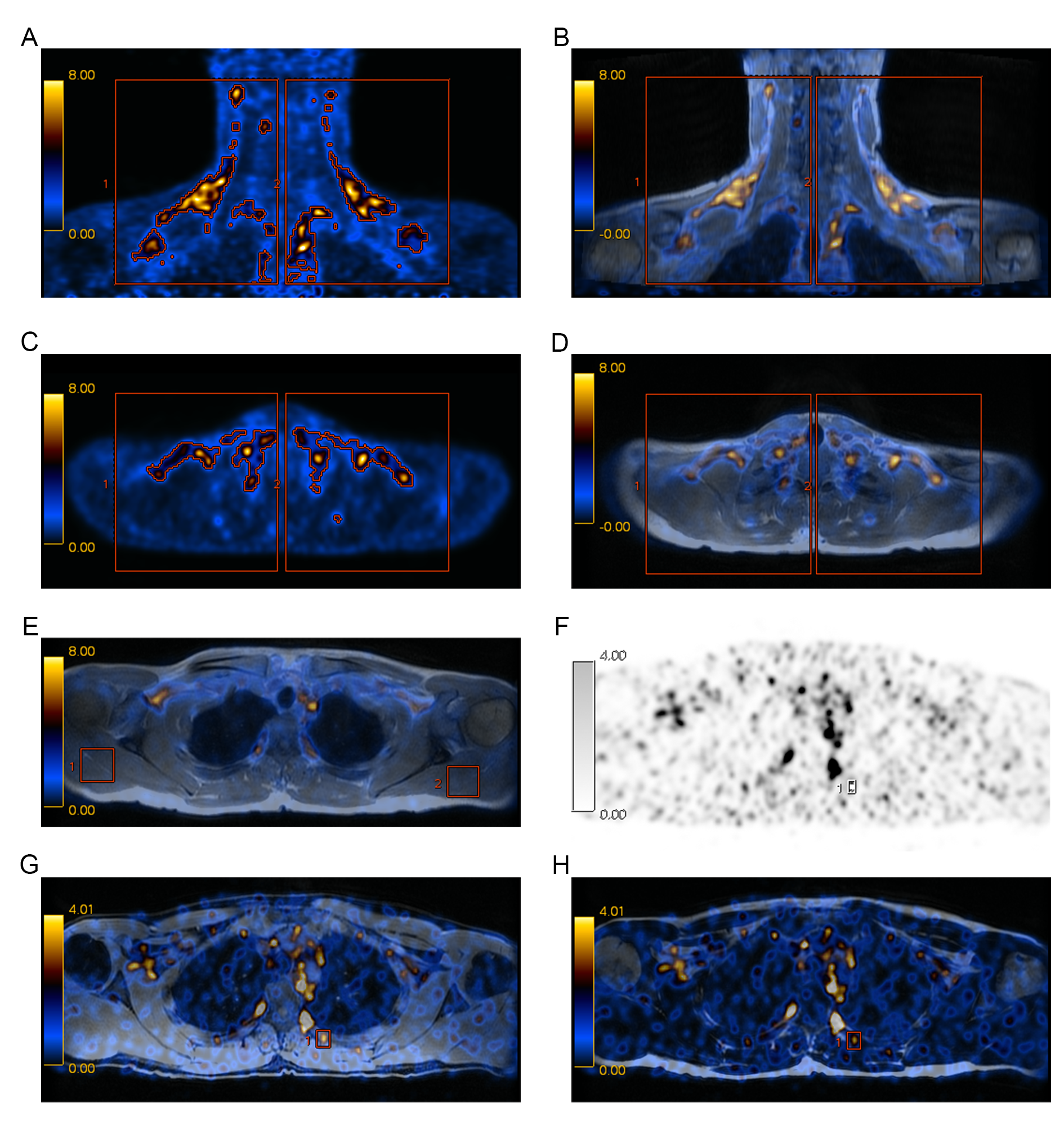


Figure S1: Examples of VOIs. (A) A coronal PET image showing two VOIs containing BAT, (B) a coronal fused PET/MR image showing the BAT, (C) an axial PET image showing two VOIs containing BAT, (D) an axial fused PET/MR image showing the BAT, (E) an axial fused PET/MR image showing VOIs for background measurements, (F) an axial 5% activity PET image showing a VOI containing an artifact, fused with Dixon based water (G) and fat (H) MR images. The colorbars indicate the SUL range (0-8 g/ml and 0-4 g/ml).

**Table S1: Background quantification**

|  | **Reference** | **35%** | **30%** | **25%** | **20%** | **15%** | **10%** | **5%** |
| --- | --- | --- | --- | --- | --- | --- | --- | --- |
| **SUL_max_** | 0.84±0.12 | 0.83±0.06  (0.54) | 0.87±0.11  (0.97) | 0.91±0.17  (0.13) | 0.97±0.11  (<0.01)* | 1.13±0.27  (<0.01)* | 1.29±0.37  (<0.01)* | 2.05±0.66  (<0.01)* |
| **SUL_mean_** | 0.41±0.04 | 0.43±0.04  (0.02)* | 0.43±0.04  (0.02)* | 0.43±0.04  (<0.01)* | 0.43±0.04  (<0.01)* | 0.42±0.05  (<0.01)* | 0.43±0.05  (<0.01)* | 0.43±0.07  (<0.01)* |
| **SUL_std_** | 0.09±0.02 | 0.10±0.02  (0.50) | 0.10±0.02  (0.15) | 0.11±0.02  (<0.01)* | 0.12±0.02  (<0.01)* | 0.14±0.02  (<0.01)* | 0.17±0.04  (<0.01)* | 0.24±0.05  (<0.01)* |
| **SUL_cov_** | 0.23±0.05 | 0.23±0.05  (0.97) | 0.25±0.05  (0.18) | 0.26±0.08  (<0.01)* | 0.28±0.06  (<0.01)* | 0.34±0.09  (<0.01)* | 0.39±0.10  (<0.01)* | 0.57±0.16  (<0.01)* |

The SUL_max_, SUL_mean_, SUL_std_ and SUL_cov_ values (median ± interquartile range) for the reference and the simulated 35% – 5% reconstructions. SUL_max_, SUL_mean_, and SUL_std_ are in g/ml. P-values are listed between brackets and * indicates a significant difference with the reference.

**Table S2: Artifact quantification**

|  | **Reference** | **35%** | **30%** | **25%** | **20%** | **15%** | **10%** | **5%** |
| --- | --- | --- | --- | --- | --- | --- | --- | --- |
| **SUL_max_** | 1.64±0.79 | 1.485±0.43  (0.35) | 1.97±0.35  (0.19) | 2.04±0.37  (<0.01)* | 2.14±0.50  (<0.01)* | 2.30±0.61  (<0.01)* | 2.78±0.76  (<0.01)* | 3.57±0.61  (<0.01)* |
| **SUL_mean_** | 0.86±0.30 | 0.95±0.30  (0.31) | 0.92±0.23  (0.19) | 0.98±0.27  (<0.01)* | 1.00±0.26  (<0.01)* | 1.14±0.26  (<0.01)* | 1.31±0.28  (<0.01)* | 1.91±0.28  (<0.01)* |

The SUL_max_ and SUL_mean_ values (median ± interquartile range) for the reference and simulated 35% – 5% reconstructions. SUL_max_ and SUL_mean_ are in g/ml. P-values are listed between brackets and * indicates a significant difference with the reference.

**Table S3: BAT quantification**

|  | **Reference** | **35%** | **30%** | **25%** | **20%** | **15%** | **10%** | **5%** |
| --- | --- | --- | --- | --- | --- | --- | --- | --- |
| **SUL_max_** | 9.30±5.83 | 9.09±5.88  (0.97) | 9.38±5.52  (>0.99) | 9.38±5.37  (0.92) | 9.88±5.88  (0.41) | 9.92±5.82  (0.34) | 9.77±6.18  (0.11) | 9.44±6.81  (<0.01)* |
| **SUL_mean_** | 2.00±0.42 | 1.99±0.41  (0.84) | 2.03±0.50  (0.15) | 1.97±0.52  (0.82) | 2.01±0.44  (0.43) | 1.95±0.45  (0.79) | 1.95±0.45  (0.53) | 1.97±0.45  (0.43) |
|  |  |  |  |  |  |  |  |  |
| **BMV** | 1.84±0.59 | 1.86±0.50  (0.61) | 1.80±0.87  (0.88) | 1.77±0.48  (0.72) | 1.80±0.67  (0.07) | 1.93±0.78  (0.02)* | 1.92±0.81  (<0.01)* | 2.19±0.66  (<0.01)* |
| **TBG** | 0.35±0.20 | 0.36±0.19  (0.81) | 0.36±0.26  (0.91) | 0.36±0.17  (0.77) | 0.36±0.18  (0.14) | 0.40±0.18  (0.02)* | 0.39±0.17  (<0.01)* | 0.44±0.19  (<0.01)* |

The SUL_max_, SUL_mean_, BMV and TBG values (median ± interquartile range) for the reference and simulated 35% – 5% reconstructions. SUL_max_ and SUL_mean_ are in g/ml, BMV in x10^2^ cm^3^ and TBG in x10^6^ mm^3^·g/ml. P-values are listed between brackets and * indicates a significant difference with the reference.
